# Exploring Recent Developments in the Manifestation, Diagnosis, and Treatment of Patients with Smith–Lemli–Opitz Syndrome: From Molecular Pathways to Clinical Innovations

**DOI:** 10.3390/ijms26146672

**Published:** 2025-07-11

**Authors:** Aleksandra Żukowska, Małgorzata Król, Patrycja Kupnicka, Katarzyna Bąk, Kamil Janawa, Dariusz Chlubek

**Affiliations:** 1 Department of Biochemistry and Medical Chemistry, Pomeranian Medical University in Szczecin, Powstańców Wlkp. 72, 70-111 Szczecin, Poland; olazukowska88@gmail.com (A.Ż.); malgorzata.krol@pum.edu.pl (M.K.); patrycja.kupnicka@pum.edu.pl (P.K.); kjanawka@interia.pl (K.J.); 2 Clinical Department of Nephrology, Transplantology and Internal Medicine, Pomeranian Medical University, 70-111 Szczecin, Poland; katarzyna.bak@pum.edu.pl

**Keywords:** cholesterol pathway, Smith–Lemli–Opitz syndrome, SLOS, DHCR7

## Abstract

Smith–Lemli–Opitz syndrome (SLOS) is a rare, autosomal recessive genetic disorder caused by mutations in the *DHCR7* gene, which encodes the enzyme responsible for the final step in cholesterol biosynthesis. Impaired enzyme function leads to cholesterol deficiency, affecting the development and function of the entire organism. The accumulation of cholesterol precursors enhances the formation of oxysterols, which are involved in the pathomechanism of neurological, ophthalmological, and vascular changes in patients. This review analyzes 53 studies published between 2020 and 2025 on the molecular mechanisms underlying the clinical features of SLOS, including cholesterol deficiency, oxysterol accumulation, and the latest diagnostic methods, including LC-MS/MS chromatography and biomarkers such as GFAP for monitoring disease progression. MRI is discussed as a supportive tool for neuroimaging, along with advances in prenatal diagnostics, such as the detection of cholesterol precursors in neonatal hair. Therapeutic options are also reviewed, with particular emphasis on cholesterol supplementation, cholic acid, and experimental treatments such as vitamin E supplementation, statin therapy, gene therapy, and liver transplantation. Current research indicates that expanding knowledge in this area not only improves patient prognosis but also provides hope for the development of effective therapies in the future.

## 1. Introduction

Smith–Lemli–Opitz syndrome (SLOS) is an autosomal recessive congenital disorder first identified in 1964 by pediatricians David W. Smith, Luc Lemli, and John Opitz from the University of Wisconsin in three unrelated male children. An alternative name for the syndrome is RSH syndrome, originally derived from the initials of the surnames of the three affected children described in early case reports. Advances in understanding the biochemical and genetic mechanisms underlying this disease have contributed significantly to improved diagnostics, treatment strategies, and overall quality of life for affected patients.

### 1.1. DHCR7 Gene Pathogenic Variants

The molecular basis of this disease lies in pathogenic variants of the 7-dehydrocholesterol reductase gene (*DHCR7*), which encodes 7-dehydrocholesterol reductase (7-DHCR), the enzyme that catalyzes the final step in cholesterol biosynthesis: the reduction of 7-dehydrocholesterol to cholesterol. These variants impair enzyme activity, leading to reduced cholesterol synthesis [1]. The *DHCR7* gene is located on chromosome 11 and consists of 12 exons [2] and eight introns [3]. In SLOS, the most commonly observed pathogenic variants are missense and nonsense variants, which result in a complete loss of enzymatic function or reduced catalytic activity. More than 90% of pathogenic variants in the *DHCR7* gene are located in exons 9, 6, and 4 [4].

**Figure 1:**
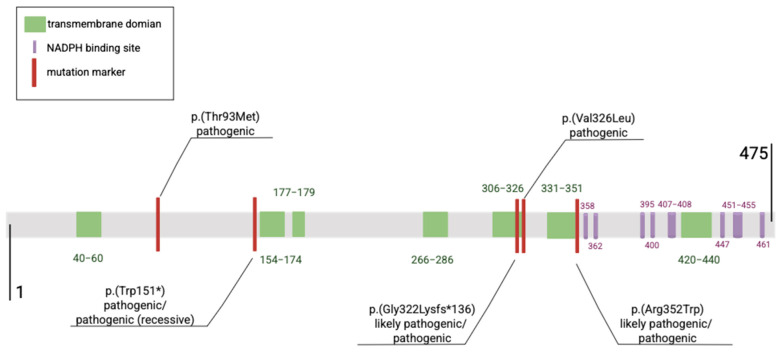
Graphical representation of the five most frequently reported DHCR7 variants and their localization within the protein structure.

To date, 180 *DHCR7* variants (reported in 1,015 cases) have been publicly documented, of which 65 are classified as pathogenic (788 cases) and 72 as likely pathogenic (142 cases). An additional 39 are of uncertain significance (42 cases) [5]. The carrier frequency of the mutated *DHCR7* gene responsible for SLOS is highest among Ashkenazi Jews living in the United States (1 in 43) and among Northern Europeans (1 in 54) [6]. Among East Asian populations, the highest carrier frequencies are found in Africans, Latinos, Ashkenazi Jews, Europeans (both Finnish and non-Finnish), and South Asians [7]. The most frequently occurring pathogenic variants include c.964-1G>C, c.452G>A, c.278C>T, c.976G>T, and c.1054C>T (Table 1, Figure 1). In northeastern and western European populations, the most common loss-of-function mutations (null mutations) are c.964-1G>C and p.Trp151X, which are associated with the most severe clinical phenotype [8]. A single case of SLOS with a biallelic *DHCR7* pathogenic variant, c.1295A>G (p.Tyr432Cys), has also been documented [9].

Not all *DHCR7* mutations impair enzyme function. Mutations occurring outside the transmembrane region or ligand-binding site do not significantly reduce cholesterol synthesis [10]. In the Leiden Open Variation Database (LOVD), 26 mutations are classified as benign or likely benign (43 cases) [11].

**Table 1:** Five most common pathogenic variants of the *DHCR7* gene and their molecular characteristics. An asterisk (*) represents a stop codon, which signals the termination of translation.

Reported	DNA Change (cDNA)	Protein	rsID	Clinical Classification	Reference
246	c.964-1G>G	p.(Gly322Lysfs*136)	-	likely pathogenic (recessive), pathogenic	[12,13]
117	c.452G>A	p.(Trp151*), p.(Trp151Ter)	rs80338854	Pathogenic, pathogenic (recessive)	[14]
96	c.278C>T	p.(Thr93Met)	rs80338853	pathogenic	[8,15,16]
50	c.976G>T	p.(Val326Leu)	rs80338859	pathogenic	[15]
31	c.1054C>T	p.(Arg352Trp)	rs80338860	likely pathogenic, pathogenic	[15]

### 1.2. SLOS Severity Score (SSS)

**Figure 2:**
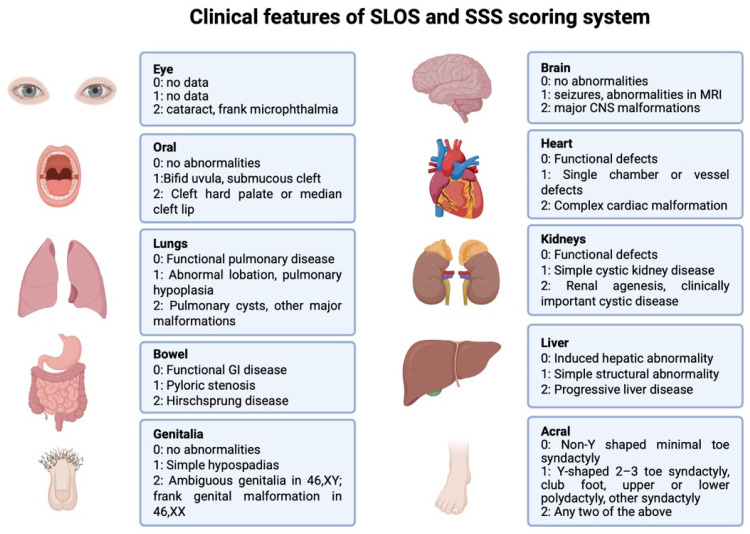
SLOS Severity Score. Types of SLOS phenotype according to SSS: mild (<20 points), typical (20–50 points), and severe (>50 points) [17,18,19] GI—gastrointestinal; MRI—magnetic resonance imaging; CNS—central nervous system.

The SLOS Severity Score (SSS) is used to assess disease severity based on malformations present in the patient. Ten organ systems are evaluated, each receiving a score from 0 to 2 depending on the observed abnormalities. Detailed scoring criteria are presented in Figure 1. The total score is summed, divided by 20, and multiplied by 100. Based on the resulting value, the SLOS phenotype is classified as mild (<20 points), classical (20–50 points), or severe (>50 points) [17,18,19] (Figure 2).

An example is the aforementioned case of a 73-year-old female patient with a biallelic pathogenic variant, who was diagnosed with a mild SLOS phenotype based on features such as syndactyly of the second and third toes on both feet, broad forehead, triangular facial shape, and short stature. The patient also suffered from amnesia, motor impairments, and recurrent hip dislocations requiring surgical intervention. Her cholesterol level was reduced (125 mg/dL), while serum 7-dehydrocholesterol (7-DHC) concentration was markedly elevated (442 μmol/L) [9].

**Figure 3:**
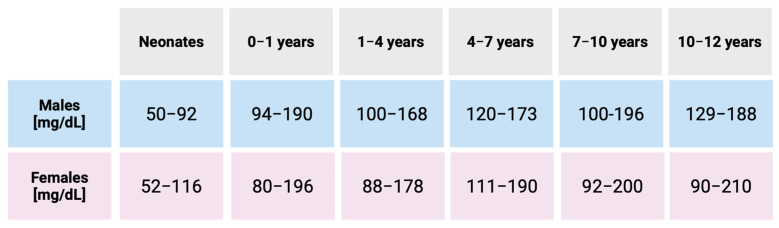
The ranges of cholesterol levels in healthy children from birth to 12 years of age, by sex [22].

The SSS score negatively correlates with blood cholesterol levels [20]. Normal lipid concentrations in healthy children are age- and sex-dependent (Figure 3) [21,22]. Newborns with severe cholesterol deficiency (below 0.35 mmol/L, equivalent to 13.53 mg/dL) typically die in the perinatal period [23]. In adults, lower total serum cholesterol levels (127.5 ± 45.1 mg/dL) are associated with poor neurological outcomes at discharge and higher in-hospital mortality following cardiac arrest [24], as well as increased mortality in advanced heart failure [25].

Selvaraman et al. evaluated mortality risk factors in a cohort of 107 individuals with SLOS, including 27 living patients and deceased individuals for whom death certificates were available. Among deceased patients, the mean SSS score was higher and the mean cholesterol concentration was lower compared to the group of living patients. Notably, age at death did not correlate with SLOS severity or baseline cholesterol and 7-DHC levels. The study also demonstrated that many individuals with SLOS reach adulthood without difficulty [26].

### 1.3. Cholesterol Deficiency and Oxysterols in the Pathogenesis of SLOS

#### 1.3.1. Cholesterol

Cholesterol is a steroid involved in myelin sheath formation [27], synaptogenesis [28], neuronal differentiation [29], synaptic integrity [30], fetal nervous system development [31], and protects against cognitive impairment [32,33]. In addition, cholesterol serves as a precursor for androgens [34] and bile acids [35], and as a component of bile, it facilitates the intestinal absorption of fat-soluble vitamins A, D, E, and K [36].

Cholesterol deficiency, especially during embryogenesis and periods of rapid growth, including central nervous system development, leads to multisystem disturbances and contributes to the clinical manifestations of 7DHCR enzyme deficiency [15]. Cholesterol plays a fundamental role in embryogenesis in all animals by activating and mediating the signal transduction of Sonic Hedgehog (SHH) proteins [37], which are essential for the development of embryonic organs, such as the neural tube, lungs, and intestines [38], and for maintaining adult tissue function [39]. Disruption of SHH signaling due to cholesterol deficiency may underlie the SLOS-related malformations and explain the inverse relationship between cholesterol levels and disease severity. 7DHCR also acts as a negative regulator of hedgehog (Hh) signaling, including SHH, by inhibiting intracellular signaling. 7DHCR dysfunction may therefore contribute to SLOS pathogenesis not only by limiting cholesterol synthesis but also by failing to suppress SHH activation [40].

Cholesterol deficiency also impairs bile acid synthesis, while the accumulation of 7- and 8-dehydrocholesterol may lead to the formation of atypical bile acids via mitochondrial sterol 27-hydroxylase (CYP27) [41]. Despite impaired endogenous cholesterol synthesis, adrenal hormone production and adrenal function remain intact in SLOS patients [42].

Cholesterol levels are primarily regulated at the step catalyzed by 3-hydroxy-3-methylglutaryl-coenzyme A (HMG-CoA) reductase [43]. Sterol regulatory element-binding proteins (SREBPs) are transcription factors that regulate lipid synthesis, particularly the biosynthesis of cholesterol and fatty acids [44]. In humans, two SREBP2 binding sites have been identified in the *DHCR7* promoter: one at position -155 and another at -55. Prabhu et al. showed that these two promoter elements synergistically activate *DHCR7* transcription, which is essential for the efficient regulation of cholesterol biosynthesis [45]. Moreover, under high intracellular cholesterol levels, HMG-CoA reductase undergoes proteolytic degradation, thereby limiting cholesterol production.

#### 1.3.2. Oxysterols

**Figure 4:**
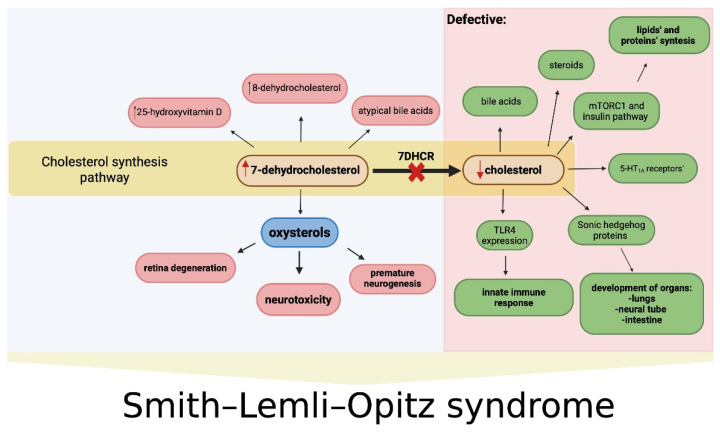
The metabolic bottleneck in Smith–Lemli–Opitz syndrome (SLOS). The deficiency of 7-dehydrocholesterol reductase (7-DHCR) leads to the accumulation of 7-dehydrocholesterol (7-DHC) and a reduction in cholesterol levels. Elevated levels of 7-DHC and its oxidized derivatives (oxysterols) contribute to neurotoxicity and premature neurogenesis. Cholesterol deficiency impairs multiple biological processes, including steroid and bile acid synthesis, 5-HT_1A_ receptor function, mTORC1 and insulin signaling, innate immune responses, and organ development. TLR4—Toll-like receptor 4; mTORC1—mammalian target of rapamycin complex 1; 5-HT_1A_—serotonin 1A receptors.

The development of clinical symptoms in SLOS results not only from impaired cholesterol synthesis but also from elevated levels of 7-DHC, one of the precursors of cholesterol. Under the influence of steroid 8-isomerase (Δ8-isomerase), 7-DHC is converted into 8-dehydrocholesterol (8-DHC) [46]. As a result, elevated levels of both 7-DHC and 8-DHC are typically observed in SLOS [47] (Figure 4).

7-DHC is a highly reactive compound that readily undergoes oxidation to form oxysterols [48]. These can be produced via free radical oxidation of 7-DHC (a nonenzymatic reaction in the presence of oxygen) [49] or through enzymatic oxidation mediated by cytochrome P450 [50]. In vitro studies on fibroblasts derived from SLOS patients and a *Dhcr7*-deficient neuroblastoma cell line have identified the major oxysterols formed from 7-DHC oxidation as 3β,5α-dihydroxycholest-7-en-6-one (DHCEO), 4α- and 4β-hydroxy-7-DHC (4-OH-DHC), 3β,5α,9α-trihydroxycholest-7-en-6-one (THCEO), 3β,5α-dihydroxycholesta-7,9(11)-dien-6-one (DHCDO), and 7-keto-cholesta-5,8-dien-3β-ol (7-kDHC) [48,51]. An additional oxysterol, 7-ketocholesterol (7-kChol), was isolated from the retinas of rats exposed during pregnancy to the DHCR7 inhibitor (trans-1,4-bis [2-dichlorobenzylamino-ethyl] cyclohexane dihydrochloride or AY9944) [52]. While 7-kChol is primarily derived from cholesterol, it can also be formed from 7-DHC via P450-mediated oxidation [53].

Oxysterols contribute to the pathogenesis of atherosclerotic cardiovascular disease (ASCVD) through pro-inflammatory, anti-inflammatory, and cytotoxic effects, partly mediated by transforming growth factor beta (TGF-β). TGF-β is a protective cytokine in ASCVD, and its signaling contributes to disease development. 7-DHC, but not cholesterol, suppresses TGF-β-stimulated luciferase activity in a concentration-dependent manner in Mv1Lu cells stably expressing the *decapentaplegic homolog 2 (Smad2)-dependent luciferase reporter gene* [54]. Furthermore, 7-DHC promotes localization of TGF-β receptors I and II (TβR-I and TβR-II) in lipid rafts/caveolae, thereby suppressing canonical TGF-β signaling, potentially increasing the risk of ASCVD [54]. 

However, at low concentrations (~0.4 μg/mL), 7-DHC enhances TGF-β-stimulated luciferase activity, similar to vitamins D2 and D3, by displacing cholesterol from resident lipid rafts/caveolae [54]. Since 7-DHC is a precursor of vitamin D, its UVB-induced photoconversion in the skin leads to elevated serum 7-DHC levels in SLOS patients. Nevertheless, no toxic effects associated with elevated 25(OH)D—such as hypercalcemia or hyperphosphatemia—have been reported in these individuals [55].

In SLOS model mice, 7-DHC was shown to suppress sterol biosynthesis posttranslationally by accelerating the proteolysis of HMG-CoA reductase. While this mechanism limits further 7-DHC accumulation, it may worsen fetal cholesterol deficiency [56]. Additionally, 7-kChol, a derivative of 7-DHC, promotes atherosclerotic plaque formation and increases cardiovascular mortality [57]. However, no studies have directly investigated the association between SLOS and the risk of ASCVD.

This review summarizes recent findings on the pathophysiology, diagnostics, and treatment of SLOS, with particular emphasis on its molecular underpinnings and clinical consequences. Detailed studies utilizing new technologies offer a better understanding of the mechanisms responsible for the clinical outcomes of the disease and guide clinicians and researchers toward identifying new therapeutic targets in SLOS management.

## 2. Methods

A literature search was conducted in the Medline (PubMed) database for relevant scientific reports using the following search terms: “Smith–Lemli–Opitz Syndrome”, “*DHCR7* mutation”, “SLOS clinical features”, “diagnostic finding in Smith–Lemli–Opitz Syndrome”, “SLOS treatment”, and their combinations. The searches were carried out from November 2024 to January 21, 2025, focusing on literature published within the past five years.

### Eligibility Criteria and Study Selection

The following inclusion criteria were applied in this narrative review: (1) clinical studies, (2) comparative studies, (3) in vivo studies, (4) in vitro studies, (5) studies published in English, (6) case reports, and (7) meta-analyses. All selected articles addressed SLOS and recent advances related to molecular pathways and clinical aspects. The accuracy, reliability, and compliance of the available reports with inclusion criteria were assessed. The following exclusion criteria were applied: (1) articles concerning SLOS but not focused on molecular pathways and/or clinical aspects, (2) review articles, (3) literature published before 2020, (4) non-peer-reviewed literature, and (5) lack of access to full-text articles (Figure 4).

**Figure 5:**
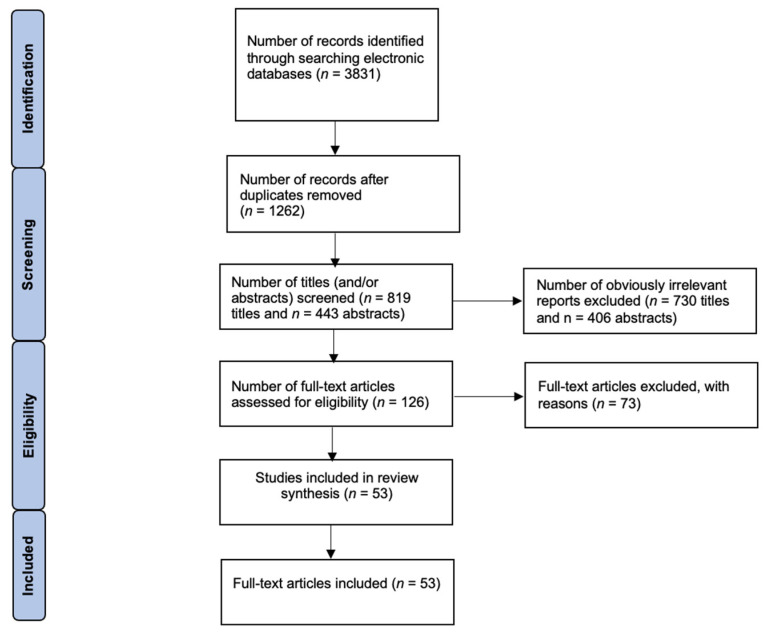
Summary of the search and selection of studies related to SLOS.

Duplicate records were removed using Zotero software (version 6.0.36). Two independent reviewers (A.Ż. and M.K.) screened the remaining articles based on titles and abstracts. Full-text articles were then assessed for eligibility. Any discrepancies were resolved through discussion with a third reviewer (P.K.). The initial search yielded 3831 articles. After removing duplicates (*n* = 1369) and completing the selection process, a total of 53 studies were included in the final analysis. The methodology flowchart is shown in Figure 5.

## 3. Update on Clinical Manifestation and Underlying Molecular Mechanisms in SLOS

### 3.1. General Features and Congenital Malformations

The clinical presentation of SLOS varies depending on serum cholesterol levels. In the prenatal period, most affected fetuses exhibit intrauterine growth restriction (IUGR) [58]. A study involving 65 Polish patients demonstrated that newborns with SLOS had lower average body length, weight, and head circumference compared to healthy controls. Higher total cholesterol levels and lower 7-DHC levels were associated with larger head circumference at birth [59].

A retrospective study of 18 patients with SLOS, including four fetuses, measured cholesterol and 7-DHC levels, documented clinical features, and analyzed *DHCR7* gene pathogenic variants. Four neonates with severe cholesterol deficiency (below 0.35 mmol/L) died in the perinatal period due to electrolyte imbalances, sepsis-like episodes, or necrotizing enterocolitis. Patients with cholesterol levels ≥ 1.7 mmol/L were diagnosed between the ages of 9 months and 25 years and exhibited a milder course. Among dysmorphic features, the most common was syndactyly of the second and third toes (13/14 patients), as well as polydactyly. Branchial arch abnormalities, including micrognathia and vision disorders, were diagnosed in 7 out of 14 patients. Additional findings included immune dysfunction, hypocalcemia, hypospadias, and cardiac defects. Thymic abnormalities were found in three of the four fetuses [23].

In an in vitro study using skin fibroblasts derived from SLOS patients, cholesterol was shown to be involved in the expression of toll-like receptor 4 (TLR4) on the cell surface. TLR4 plays a key role in detecting Gram-negative bacteria via lipopolysaccharides (LPS). Cholesterol deficiency reduced TLR4 expression, impairing the innate immune activation and potentially increasing susceptibility to infections in SLOS patients [60].

As patients grow older, they may develop craniofacial dysmorphisms (e.g., hypertelorism and a flattened, upturned nose). Recent case reports have expanded the list of known congenital malformations associated with SLOS, including heart defects (ventricular septal defect, atrial septal defect), renal malformations, hypospadias [61], central nervous system anomalies, and infantile hypertrophic pyloric stenosis [62].

### 3.2. Neurological Disorders

Ninety percent of rare childhood disorders have major neurological effects [63]. In SLOS, the clinical picture often includes psychomotor delay and intellectual disability. Observational case series in recent years reported cognitive and adaptive impairments in affected individuals, many of whom meet the criteria for autism spectrum disorder (ASD) [64,65]. However, a retrospective data analysis by Kaub et al. suggested that additional biochemical testing for SLOS (e.g., 7-DHC measurement) is not warranted in individuals already diagnosed with autism or ASD [66], as SLOS accounts for only a small fraction of autism spectrum cases [66].

Children with SLOS frequently experience delays in reaching developmental milestones such as walking, speech, and toilet training. An observational study showed a negative correlation between cerebrospinal fluid levels of 7-DHC, 8-DHC, and cholesterol and functional abilities in patients [64].

Moreover, disruptions in cholesterol metabolism and accumulation of its precursors may contribute to depressive symptoms [67]. Studies have found that individuals with moderate to severe depression exhibit reduced cholesterol and elevated 7-DHC levels—findings consistent with the lipid profile of SLOS. A post-mortem retrospective case–control analysis revealed that 7-DHC levels in the brains of patients treated with trazodone were over ten times higher than in untreated individuals. Trazodone, a psychotropic drug used primarily to treat depression, appears to inhibit 7DHCR and may be responsible for the observed sterol imbalance [68]. Caution is therefore advised when prescribing trazodone to individuals with 7DHCR deficiency, and treatment regimens should be adjusted accordingly.

Emerging data also indicate the presence of hearing loss in SLOS. In a cross-sectional observational study of 32 patients, hearing impairment was detected in more than 50% (17 individuals), with conductive (45%), sensorineural (34.5%), and mixed types reported. Audiological brainstem response testing in 8 of 21 patients revealed abnormalities consistent with retrocochlear hearing loss [69].

Due to the rarity of the condition and variability in disease severity, the statistical power of these studies is limited. Continued research is needed to confirm these associations and improve understanding of neurological manifestations in SLOS.

#### Molecular Aspects

The neurological symptoms described above are linked to molecular disruptions caused by reduced cholesterol levels and elevated concentrations of oxysterols. In a preclinical model, zebrafish with pathogenic *DHCR7* variants exhibited reduced neuronal numbers, abnormal myelin formation, disorganized synaptic clefts, and elevated dopamine and norepinephrine levels—manifesting behaviorally as attention-deficit/hyperactivity disorder (ADHD)-like traits and reduced aggression [70].

In experimental studies using human induced pluripotent stem cells (hiPSCs) derived from SLOS patient fibroblasts and mouse cortical progenitor cells, pathogenic variants of *DHCR7* impaired cortical development and disrupted self-renewal of neural precursors. Oxysterols derived from 7-DHC, such as DHCEO and 4α-OH-7DHC, have been implicated in these abnormalities. DHCEO interacted with the glucocorticoid receptor (GR), triggering activation of the receptor tyrosine kinase (RTK)-mediated mitogen-activated protein kinase–extracellular signal-regulated kinase CCAAT/enhancer binding protein (MEK–ERK–C/EBP) signaling pathway. MEKs are kinases that phosphorylate tyrosine and/or threonine residues in regulatory domains of ERKs, leading to ERK activation [71]. Through RTKs, MEK is activated, followed by ERK, enabling the proper development of cortical precursor cells and subsequent neuronal differentiation [72].

In an SLOS in vitro model, RTK activation led to abnormal, premature neurogenesis, potentially increasing the risk of neurological disorders and contributing to the formation of aberrant synaptic connections and impaired neural development. Notably, this process can be reversed by GR inhibitors or antioxidant treatment [73].

Another study conducted on fibroblasts from SLOS patients and induced pluripotent stem cells (iPSCs) emphasized that 7-DHC—not cholesterol—was responsible for increased βIII-tubulin, microtubule-associated protein 2 (MAP2), and glial fibrillary acidic protein (GFAP) synthesis and overexpression of tubulin beta 3 class III (*TUBB3*), *MAP2*, *GFAP,* and synaptophysin (*SYP*) genes, contributing to neural differentiation [74]. Furthermore, 7-DHC inhibited rosette formation, disrupting early neurodevelopment during embryogenesis [75]. It also blocked the β-catenin-dependent wingless/integrated (Wnt)/β-catenin signaling pathway, which is essential for blood–brain barrier development, CNS vascularization, and pre- and postnatal neuronal maturation [76].

While these findings provide valuable insights into SLOS pathophysiology, further research is needed, and it remains uncertain to what extent they can be directly extrapolated to the complex physiology of a human organism.

### 3.3. The Visual System

Several case reports have documented ophthalmic complications in patients with SLOS, including glaucoma [77] and cataracts [78]. Among individuals exhibiting self-injurious behaviors, which are common in SLOS, 54% develop traumatic rhegmatogenous retinal detachment, resulting in vision loss [79]. Recently, López-Cañizares and Al-khersan reported the case of a 15-year-old boy with SLOS who presented with retinal detachment, retinal avascularity, vitreous hemorrhage, and glaucoma, all resulting from self-inflicted trauma [80].

#### Molecular Aspects

In vitro studies have shown that visual disturbances in SLOS are also driven by the toxic effects of oxysterols on retinal cells [81,82]. Oxidation products of 7-DHC have been directly implicated in degenerative changes within the retina. In a preclinical study using three retinal cell populations—Müller glia-derived cells, photoreceptor-derived cells (661W), and normal diploid monkey retinal pigment epithelium (mRPE) cells—photoreceptor-derived cells were the most sensitive to the cytotoxic effects of 7-kDHC, 5,9-endoperoxy-cholest-7-en-3β,6α-diol (EPCD), DHCEO, and 4β-hydroxy-7-dehydrocholesterol (4HDHC). These oxysterols inhibited cell proliferation and reduced retinal cell viability [81]. In another in vitro experiment using pluripotent retinal pigment epithelial (RPE) cells, differences in the expression of RPE-specific markers and epithelial–mesenchymal transition (EMT) markers were found between *DHCR7*-mutated and wild-type cells. The cells were divided into three groups: severe SLOS genotype, mild SLOS genotype, and normal genotype. Cellular changes were closely analyzed across the three groups, including morphology, structure, integrity, and lipid droplet accumulation. In severe SLOS genotype cells, elevated levels of vimentin degradation products and reduced levels of cellular retinaldehyde-binding protein (CRALBP) and ezrin were observed compared to the other cell groups. In both cell groups with genotypes associated with SLOS, increased expression of α-smooth muscle actin (SMA), retinal pigment epithelium-specific 65 kDa protein (RPE-65), E-cadherin, and ezrin was detected, indicating cellular remodeling and a reversion to a mesenchymal phenotype. Additionally, fibroblasts from the severe and mild SLOS genotype groups also showed enhanced accumulation of lipid droplets [83].

Beyond morphological changes, oxysterols in retinal cells also affect gene transcription. In another experimental study, conducted on 661W cells (a retinal ganglion precursor-like cell line), EPCD and 7-kCHOL exposure altered colony morphology and reduced cell number. EPCD-treated cells showed twice as many differentially expressed genes (DEGs) compared to those treated with 7-kCHOL, cholesterol, or hydroxypropyl-β-cyclodextrin. Many DEGs were unique to the EPCD treatment group and were involved in the regulation of ER stress, which, when excessively stimulated, can lead to cell death. Upregulation of ER stress-related genes was associated with a reduction in cell viability in oxysterol-treated colonies [82].

Although these experimental findings offer important insights into the molecular mechanisms of retinal dysfunction in SLOS, validation in human models is needed to confirm their physiological relevance.

### 3.4. Other Systemic Molecular Implications

Cholesterol is essential for the proper functioning of several signaling pathways, including the mammalian target of rapamycin complex 1 (mTORC1), insulin signaling, and the correct trafficking of serotonin 1A receptors (5-HT_1A_) to the cell membrane. The 5-HT_1A_ receptor has inhibitory potential and regulates multiple physiological processes such as blood pressure, heart rate, pain perception, and cognitive functions, including memory, mood, impulsivity, and sociability. In an in vitro study using cells with an SLOS genotype, a reduced number of 5-HT_1A_ receptors on the cell surface was observed due to receptor internalization [84].

Activation of mTORC1 by insulin promotes anabolic pathways such as the synthesis of proteins, lipids, and DNA—processes crucial for proper organismal development [85]. Interestingly, in SLOS-affected cells, an alternative lysosome-dependent pathway for 5-HT_1A_ receptor expression on the cell membrane was identified [84].

Another molecule of interest is inhibitory factor 1 (IF1), whose levels positively correlate with high-density lipoprotein (HDL) cholesterol and negatively with triglycerides [86]. IF1 has also been identified as a biomarker associated with the risk of type 2 diabetes [87]. In one case report, circulating IF1 was detected in the serum of two pediatric dizygotic twins with SLOS who were receiving dietary cholesterol supplementation, suggesting that IF1 may play a role in maintaining cholesterol homeostasis and could serve as an additional clinical marker of disease progression [88].

Moreover, a cross-sectional comparative survey reported that children with SLOS brush their teeth significantly less frequently than healthy controls and have poorer oral hygiene and lower utilization of dental care services. These findings indicate the need for targeted oral hygiene education and dental care in this population. When combined with impaired innate immunity—resulting from reduced expression of the TLR4 [60]—poor oral hygiene may increase the risk of dental disease-related complications such as malnutrition or infective endocarditis [89,90].

## 4. Update on SLOS Diagnostics and Disease Progression Monitoring

### 4.1. Basic Diagnostics

The diagnosis of SLOS is typically based on the patient’s biochemical profile, namely the presence of 7-DHC and 8-DHC and reduced cholesterol levels in plasma or other tissues [91]. Confirmation requires the identification of pathogenic variants in the DHCR7 gene [9]. One of the earliest diagnostic methods, developed in the 1990s, was UV spectrometry for detecting 7-DHC in serum [92]. Time-of-flight secondary ion mass spectrometry (TOF-SIMS) was also applied to diagnose newborns by analyzing filter paper plasma samples [93].

In subsequent years, gas chromatography–mass spectrometry (GC-MS) was introduced, allowing for rapid and effective SLOS diagnosis from small blood samples, including those from neonates [94]. In recent years, liquid chromatography–tandem mass spectrometry (LC-MS/MS) has been refined as a technique that enables rapid analysis of serum samples and allows both qualitative and quantitative identification of sterols. LC-MS/MS can also differentiate SLOS from other congenital disorders of cholesterol metabolism, such as sitosterolemia [95].

Both GC-MS and LC-MS/MS are used to measure cholesterol and oxysterol levels. However, GC-MS requires additional sample preparation, making it more time-consuming and increasing the risk of laboratory error. In a study comparing the two methods for cholesterol and dehydrocholesterol quantification, GC-MS yielded significantly lower concentrations than LC-MS/MS, indicating lower sensitivity. For this reason, LC-MS/MS is increasingly preferred for sterol quantification [96,97].

More recently, LC-MS/MS has been presented as a method capable of simultaneously differentiating oxysterols and bile acids from a single serum sample, providing precise concentration measurements. This may allow more accurate monitoring of SLOS progression, since both cholesterol and bile acid levels are reduced in affected individuals [98].

### 4.2. Glial Fibrillary Acidic Protein (GFAP)

In addition to measuring cholesterol, 7-DHC, 8-DHC, and oxysterols in patient serum, glial fibrillary acidic protein (GFAP) levels can also be assessed in the cerebrospinal fluid (CSF) of individuals with SLOS. GFAP is an intermediate filament produced by mature astrocytes [99]. In clinical settings, serum GFAP serves as a biomarker for diagnosing and assessing the severity of neurodegenerative diseases such as multiple sclerosis, and it is increasingly recognized as a potential blood-derived biomarker for diagnosing and monitoring brain and spinal cord disorders, including traumatic brain injury [100,101,102,103].

Studies have shown that GFAP levels correlate with disease severity and progression in neurodegenerative disorders [104,105]. In a preclinical study using transgenic mice with a *DHCR7* pathogenic variant, impaired cholesterol synthesis affected microglial and astrocyte function. Microglial activation led to astrocyte hypertrophy, increased GFAP synthesis, and impaired glutamate responsiveness and calcium signaling. The study demonstrated that cholesterol homeostasis is essential for normal astrocyte and microglial function, and its disruption contributes to neurological abnormalities seen in SLOS [106] and Alzheimer’s disease [107].

Retrospective case–control studies have found that SLOS is associated with excessive astrocyte activation—known as astrogliosis—and neuroinflammation, resulting in elevated GFAP levels in CSF. Therefore, GFAP concentration may serve as an indicator of current disease progression [108,109]. Additionally, a case study suggested that a decrease in GFAP levels correlates with reduced astrocyte activity in the CNS, making GFAP a useful marker for evaluating treatment effectiveness and identifying the regression of specific symptoms and behaviors [110].

### 4.3. Neuroimaging Techniques

Neuroimaging plays an important role in the diagnosis and severity assessment of SLOS. A clinical study involving 55 patients with confirmed SLOS demonstrated that magnetic resonance imaging (MRI) can detect structural abnormalities in the CNS related to disease progression. A total of 173 brain MRI scans were performed, revealing abnormalities in 96% of cases, most commonly involving midline brain structures. The most frequent finding was a septal defect affecting the separation of the anterior horns of the lateral ventricles (observed in 76% of patients), while 69% of patients had abnormalities of the corpus callosum. The study showed a strong correlation between the extent of brain damage (referred to as the brain severity score) and other clinical markers of SLOS severity. Higher SSS, elevated baseline 7-DHC levels, and lower baseline cholesterol concentrations were associated with more severe brain abnormalities [111].

An experimental study by Li et al. investigated cholesterol, 7-DHC, and its oxysterol accumulation in brain regions in *DHCR7*-depleted mice. The experiment, conducted on transgenic mice with a *DHCR7* null mutation (corresponding to the severe SLOS phenotype), utilized matrix-assisted laser desorption ionization mass spectrometry imaging (MALDI-MSI), a technique that combines MALDI with mass spectrometry and enables the identification of cell types as well as the analysis of proteins, lipids, and metabolites at single-cell resolution. MALDI-MSI allowed spatial mapping of cholesterol and its precursors in the mouse brain. In their animal model, Li et al. found that neurotoxic oxysterols most frequently and abundantly accumulated in the corpus callosum, outer cortical layers, and periventricular regions—areas critical for interhemispheric communication, motor coordination, and cognitive processes, all of which are impaired in SLOS [112].

### 4.4. Prenatal and Perinatal Diagnostics

Both invasive and non-invasive approaches are used in the prenatal diagnosis of SLOS. Invasive methods include measuring cholesterol and 7-DHC levels in amniotic fluid or chorionic villus samples using GC-MS [113]. An experimental study involving 21 pregnancies showed that identifying *DHCR7* pathogenic variants from these samples is a highly effective diagnostic approach [114].

The level of GFAP can also be determined in amniotic fluid. While GFAP is used in the prenatal diagnostics of other conditions [115], and its alterations in cerebrospinal fluid may help monitor SLOS progression, no current studies support its use in the prenatal diagnosis of this syndrome.

Given the risk of miscarriage associated with invasive procedures [113], efforts have been made to develop non-invasive diagnostic methods. A large multicenter trial by Shackleton et al. demonstrated that urinary measurement of 7-dehydropregnanetriol (7-PT), pregnanetriol (PT), 8-dehydropregnanetriol (8-PT), dehydroestriol, and estriol between gestational weeks 14 and 22 offers high diagnostic accuracy for SLOS [116].

Ultrasound imaging (US) is also a helpful tool in prenatal SLOS detection. It can reveal IUGR, polydactyly, and CNS and cardiac malformations—especially involving the corpus callosum [58]. However, its diagnostic specificity is limited because similar ultrasound abnormalities can occur in other syndromes such as desmosterolosis [117], Patau syndrome, or Edwards syndrome [118]. Therefore, a comprehensive diagnostic approach should integrate both laboratory and imaging data to support the clinical evaluation.

**Figure 6:**
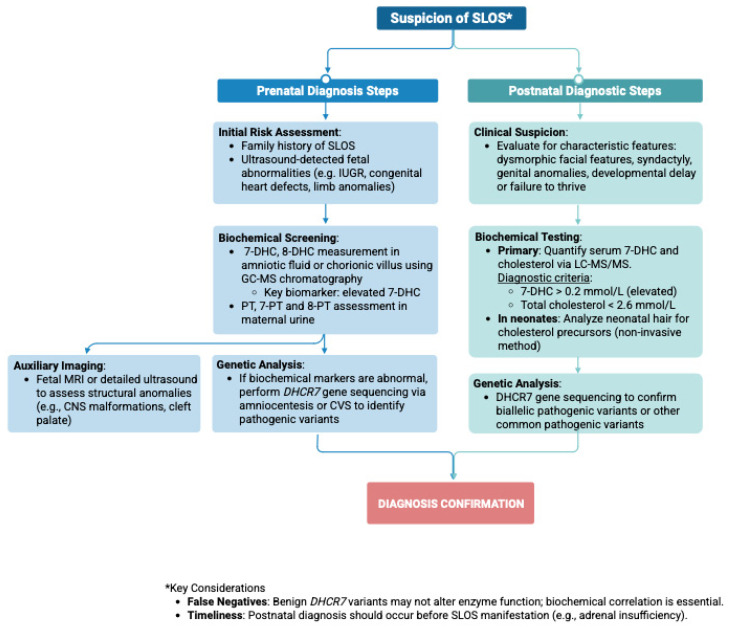
Flowchart depicting the diagnostic pathway from clinical suspicion to confirmation of SLOS across prenatal and postnatal periods. 7-DHC—7-dehydrocholesterol; 8-DHC- 7-dehydrocholesterol; LC-MS/MS—liquid chromatography–tandem mass spectrometry; MRI—magnetic resonance imaging; CNS—central nervous system; CVS—chorionic villus sampling; IUGR—intrauterine growth restriction.

Recent studies have proposed the measurement of characteristic cholesterol precursors in neonatal hair as a potential diagnostic method. While promising, this approach still faces technical limitations and requires further refinement. Nevertheless, despite its current challenges, measuring 7-DHC and cholesterol levels in hair has been identified as a reliable diagnostic tool for the detection of SLOS [119]. A detailed protocol emphasizing the integration of biochemical, genetic, and clinical data for definitive SLOS diagnosis is provided in Figure 6.

## 5. Treatment

### 5.1. Exogenous Cholesterol Supplementation

One of the initial therapeutic approaches in SLOS involves exogenous cholesterol supplementation to compensate for its deficiency. Supplementation has been associated with clinical improvement, notably in psychomotor development, even when baseline cholesterol levels vary [80]. Cholesterol intake may also lower serum concentrations of 7- and 8-DHC in patients with SLOS through negative feedback inhibition of HMG-CoA reductase [120]. However, this effect is not consistent; in some cases, elevated 7-DHC levels persist for years after initiating cholesterol therapy [121].

Importantly, unlike oxysterols [122,123], cholesterol cannot cross the blood–brain barrier and must instead be synthesized locally within the CNS by neurons, astrocytes, and oligodendrocytes [124]. Thus, exogenous cholesterol supplementation does not affect CNS function [125].

### 5.2. Statin Therapy

Statins inhibit HMG-CoA reductase, preventing the conversion of HMG-CoA to mevalonate and thereby reducing the overall synthesis of sterols, including 7-DHC [126]. In a pilot study involving 23 patients with SLOS, Wassif and Kratz evaluated the therapeutic potential of simvastatin. Patients received simvastatin in two 12-month treatment periods: 0.5 mg/kg/day for the first 6 weeks and 1.0 mg/kg/day for the remaining 46 weeks. These treatment phases were separated by a 2-month washout period. Following therapy, a reduction in serum and CSF 7-DHC levels was observed, alongside clinical improvement in patient behavior [121].

However, a 2022 review summarizing studies on statin therapy in SLOS found no current evidence supporting its impact on survival or quality of life in affected individuals [127]. Furthermore, evidence for the efficacy of statins in alleviating neurobehavioral symptoms remains limited.

Nonetheless, a clinical trial involving 39 SLOS patients indicated that simvastatin administration reduced GFAP levels in both serum and CSF [108]. In one case, a 25-day-old male patient with SLOS and complex congenital malformations received simvastatin at 0.5 mg/kg/day for 6 weeks, followed by 1.0 mg/kg/day for 12 months. Clinical follow-up showed improved condition and good treatment tolerance [110].

Despite these promising outcomes, further studies involving larger patient cohorts are necessary to thoroughly evaluate the therapeutic scope, long-term effects, and applicability of statins in adult SLOS patients.

### 5.3. Cholic Acid Supplementation

A recent pilot study investigated the effects of cholic acid supplementation on cholesterol and its precursors in patients with SLOS. Cholic acid, a primary bile acid synthesized in the liver, is used therapeutically in patients with inborn errors of bile acid biosynthesis [128]. In the study, patients received cholic acid at a dose of 10 mg/kg/day for 2 months. GC-MS was used to measure serum cholesterol, 7-DHC, and 8-DHC, while LC-MS was used to assess oxysterol levels. Results showed an increase in serum cholesterol levels following treatment. However, no changes were observed in the concentrations of 7-DHC or 8-DHC. These findings suggest that cholic acid should not be used as monotherapy but may provide added benefits in combination with exogenous cholesterol and antioxidant therapy [47].

### 5.4. Antioxidants

Antioxidants appear to be a powerful tool in combating SLOS. In an in vitro experiment conducted on fibroblast cultures derived from SLOS patients, as well as in vivo on pregnant mice, it was demonstrated that antioxidants—particularly vitamin E—prevent the accumulation of oxysterols in fibroblasts and in the liver and brain tissues of newborn mice [129].

A prospective interventional pilot study evaluated the effects of long-term vitamin E supplementation in individuals with SLOS. The study examined serum levels of vitamins A and E, 7-DHC, 8-DHC, cholesterol, and behavioral changes. All patients had vitamin E concentrations that were either low–normal or below the reference range. Participants received vitamin E for 3 years: 230 mg daily for those aged 4–10 years and 2 × 230 mg for those over 10 years, administered with food or orally. After 12 months, the dose was reduced by 50% in two patients. After three years, three patients with initially low ratios of (7-DHC + 8-DHC) to total cholesterol responded clinically to the supplementation, showing positive behavioral changes. The study concluded that vitamin E supplementation may be beneficial in SLOS, but the response depends on baseline cholesterol and precursor levels [130]. Further validation in larger patient cohorts is necessary.

### 5.5. Liver Transplantation

A recently reported case involved a 19-month-old child with SLOS who developed liver cirrhosis and underwent liver transplantation. Thirty-four months post-transplant, the patient showed improvements in both motor and behavioral development. Although liver disease occurs in only 2.5–16% of individuals with congenital cholesterol synthesis disorders, this case demonstrated that liver transplantation can significantly reduce the clinical manifestations of SLOS. The authors suggested considering this option early in the disease course based on clinical presentation and prognosis [131].

### 5.6. Gene Therapy

A study conducted a decade ago explored the potential of gene therapy as a treatment strategy. In genetically modified mice with a *DHCR7* pathogenic variant mimicking the SLOS phenotype, a single intracerebral administration of a viral vector carrying the wild-type *DHCR7* gene resulted in reduced levels of 7-DHC in the brain and spinal cord two months post-treatment [132]. However, this line of research has not been pursued further, and no clinical studies using gene therapy have been conducted in human patients to date.

## 6. Discussion

SLOS is the clinical manifestation of pathogenic variants in the *DHCR7* gene, leading to impaired cholesterol synthesis and accumulation of toxic oxysterols. It is characterized by disruptions at the embryonic stage, malformations of the central nervous system, and subsequent loss of function, as well as atrial and atrioventricular septal defects, renal anomalies, and holoprosencephaly [61].

Advances in science have deepened our understanding of the biochemical and molecular mechanisms underlying the disease and led to the development of increasingly precise diagnostic methods. This review has outlined key advances across various fields of medicine concerning cholesterol homeostasis and metabolism disorders in patients with SLOS, while also pointing to ongoing challenges that remain to be addressed.

Rare diseases such as SLOS exhibit considerable clinical variability, making them difficult to diagnose [133]. A genotype–phenotype correlation refers to the association of a specific genetic mutation with a defined clinical phenotype [134]. In SLOS, this correlation may vary across populations [135] and can be confounded by overlap syndromes in which patients present with additional comorbidities [136]. Early diagnosis is particularly difficult in mild SLOS cases due to non-specific phenotypic features. Moreover, genotype–phenotype correlation studies are hindered by clinical heterogeneity, small patient numbers, and the global dispersion of affected individuals [136]. Broader clinician access to publicly available genotype–phenotype databases for rare diseases—including SLOS—could facilitate earlier and more accurate diagnosis [137].

Another promising avenue is the integration of next-generation phenotyping and the TRANSLATE NAMSE model into interdisciplinary rare disease diagnostics. These approaches combine exome sequencing with artificial intelligence-based facial phenotyping, offering improved diagnostic outcomes for individuals with inconclusive results and enabling earlier identification of ultra-rare genetic conditions [138,139].

SLOS symptoms range from severe or lethal congenital malformations to subtle neurobehavioral abnormalities. On the molecular and cellular levels, neurological deficits in SLOS are associated with impaired myelination, synaptic anomalies, and neurotransmitter imbalances [70]. Interestingly, patients with ASD—who share certain neurobehavioral features with SLOS patients—also exhibit altered cholesterol metabolism, neuroinflammation, oxidative stress, and impaired myelination and synaptogenesis [140], suggesting possible mechanistic overlap. However, studies have shown that *DHCR7* pathogenic variants are rare among ASD patients, and isolated autism is not an indication for biochemical screening for SLOS [66].

Further research is needed to identify genetic and environmental modifiers that influence disease progression in SLOS. One observational study indicated that certain environmental factors, combined with disease severity and disrupted cholesterol biosynthesis, may predispose individuals with SLOS to premature death. These environmental factors, however, remain poorly defined and warrant future investigation [26].

### 6.1. Diagnostics

In prenatal diagnostics of genetic abnormalities, there has been a shift away from invasive procedures—due to their associated risk of miscarriage—towards non-invasive prenatal testing (NIPT) [141]. In the context of SLOS, such testing may include measurement of urinary levels of 7-PT, PT, 8-PT, dehydroestriol, and estriol in pregnant women, as well as conventional ultrasound imaging.

However, ultrasound lacks diagnostic specificity. Many congenital malformations observed in SLOS, such as hypospadias and cleft palate, are also present in other genetic syndromes, including Toriello–Carey syndrome [142] and Schilbach–Rott syndrome [143], as well as in fetuses and infants born to mothers with a history of fentanyl use during pregnancy. It has been hypothesized that long-term fetal exposure to fentanyl may disrupt cholesterol biosynthesis, leading to reduced cholesterol levels and elevated concentrations of its precursors (7-DHC and 8-DHC) in the serum of affected newborns [144].

Due to the rarity of SLOS and the current lack of an established effective treatment, prenatal diagnosis is not a clinical priority. Nonetheless, it remains essential to include SLOS in the differential diagnosis, not only for other syndromic disorders but also for conditions resulting from prenatal narcotic exposure.

The combination of advanced biochemical and molecular laboratory analyses with imaging techniques currently represents the most effective approach for diagnosing SLOS. Contemporary laboratory techniques allow for precise assessment of patient samples for cholesterol, its precursors, and their derivatives [94,95,98]. Measurement of GFAP in CSF [106] and serum [109] provides an additional tool for monitoring disease severity and progression.

Recent technological advances have also enabled the use of MALDI-MSI for identifying and spatially visualizing the distribution of cholesterol and its precursors in tissues, contributing to a more accurate understanding of SLOS pathophysiology [112].

### 6.2. Treatment

The current primary pharmacotherapeutic strategy for SLOS is exogenous cholesterol supplementation, making a high-cholesterol diet the recommended form of nutritional management [145]. Although the efficacy of statin use in SLOS was previously disputed [146], recent studies suggest that simvastatin therapy may improve the clinical picture in affected patients [110,121]. Positive outcomes have also been observed with vitamin E [130] and cholic acid supplementation [47], although these findings need confirmation in larger, well-defined study populations [130]. Further investigation is warranted to assess the effectiveness of combination therapies (e.g., statins, cholic acid, and vitamin E).

Emerging evidence indicates that liver transplantation in the context of cholesterol biosynthesis defects may halt neurological deterioration [131,147]. However, this option should be considered with careful evaluation of the full clinical picture, patient age, and a comprehensive risk–benefit analysis.

In managing the consequences of *DHCR7* pathogenic variants, it is essential to consider the mechanisms of action and side effects of pharmacological agents. Substances that alter cholesterol levels and metabolism, such as trazodone [68], may be particularly harmful to patients with SLOS and should be prescribed with caution.

As with other rare diseases, available therapies for SLOS primarily alleviate symptoms and improve quality of life but do not provide a cure or comprehensive disease modification [148,149]. Management typically includes rehabilitation and symptom-specific care coordinated by interdisciplinary teams of specialists [150,151]. The future of medical care is increasingly focused on personalized diagnostics, prognosis, and treatment—particularly through the identification of biomarkers that can predict treatment response [152,153]. Such biomarkers may also reflect disease progression and severity, as shown by blood-based biomarkers in muscular dystrophies [154].

The “N-of-1 trials” model, which involves personalized experimental treatment trials in a single patient, has been proposed as a method to optimize therapy in rare diseases. Applying such an approach to SLOS could improve clinical outcomes [155].

In scientific research, patient-derived cells offer a valuable platform for exploring disease mechanisms, complementing data from transgenic animal models. A deeper understanding of the molecular alterations responsible for the clinical manifestations of SLOS may contribute to the development of novel therapeutic strategies. These may include inhibition of GR activation or prevention of oxysterol accumulation using antioxidants to counteract premature neurogenesis phenotypes associated with 7-DHCR deficiency [73].

In vitro studies also laid the groundwork for potential gene therapy. Although gene therapy for SLOS has not yet been tested in clinical trials, preclinical studies in animal models have demonstrated improvements in cholesterol levels in serum and liver tissue following treatment [132,156], indicating potential in this therapeutic direction. Encouraging advances in gene therapy for other rare disorders, such as metachromatic leukodystrophy [157], could serve as an impetus for pursuing similar approaches in SLOS. However, the rarity of the condition poses a major challenge for conducting randomized controlled trials.

## 7. Conclusions

In recent years, significant progress has been made in understanding the pathomechanism of SLOS and the potential causes of premature death among affected individuals. Research has elucidated key mechanisms underlying the development of abnormalities in SLOS and enabled the implementation of more precise diagnostic strategies through the use of analytical techniques such as GC-MS and LC-MS/MS. These methods allow not only for accurate quantification of cholesterol but also for the identification of its precursors and confirmation of oxysterol accumulation—an essential criterion for the diagnosis of SLOS.

Prenatal diagnostic options should also be considered, such as the measurement of 7-PT, PT, 8-PT, dehydroestriol, and estriol in maternal urine. In the assessment of disease severity and progression, monitoring GFAP levels in the CSF may serve as a useful biomarker.

Recent studies have reported the beneficial effects of statin therapy and antioxidant supplementation in SLOS. However, the efficacy of these interventions requires further validation in larger patient cohorts, since existing research is limited by small sample sizes, lack of control groups, and reliance on preclinical or pilot data, which collectively constrain the strength and generalizability of their conclusions. Further research, including well-designed, translational studies in human populations, is essential to enable robust and clinically relevant insights into SLOS and its management.

At present, no definitive treatment exists for SLOS, and current approaches remain primarily symptomatic. Gene therapy presents a promising future direction, though its development demands substantial financial, personnel, and time investment. Patients—especially those with milder SLOS phenotypes—can reach adulthood, and even advanced age, with appropriate treatment and supplementation.

Raising awareness of SLOS among healthcare professionals is critical, particularly regarding its subtle clinical presentations. Furthermore, the development and implementation of effective diagnostic and screening tools in resource-limited settings are essential to ensure timely diagnosis, initiate appropriate treatment, and ultimately improve clinical outcomes for patients with SLOS.

## Abbreviations

The following abbreviations are used in this manuscript:

25(OH)D	25-hydroxyvitamin D
4HDHC	4β-hydroxy-7-dehydrocholesterol
4-OH-DHC	4α- and 4β-hydroxy-7-DHC
5-HT_1A_	Serotonin 1A receptors
7-DHC	7-dehydrocholesterol
7-DHCR	7-dehydrocholesterol reductase
7-kChol	7-ketocholesterol
7-kDHC	7-keto-cholesta-5,8-dien-3β-ol
7-PT	7-dehydropregnanetriol
8-DHC	8-dehydrocholesterol
8-PT	8-dehydropregnanetriol
ASCVD	Atherosclerotic cardiovascular disease
ASD	Autism spectrum disorder
C/EBP	CCAAT/enhancer binding protein
CNS	Central nervous system
CRALBP	Cellular retinaldehyde-binding protein
CSF	Cerebrospinal fluid
CYP27	Sterol 27-hydroxylase
DEGs	Differentially expressed genes
DHCDO	3β,5α-dihydroxycholesta-7,9(11)-dien-6-one
DHCEO	3β,5α-dihydroxycholest-7-en-6-one
DHCR7	7-dehydrocholesterol reductase gene
EMT	Epithelial–mesenchymal transition
EPCD	5,9-endoperoxy-cholest-7-en-3β,6α-diol
ER	Endoplasmic reticulum
ERK	Extracellular signal-regulated kinase
GC-MS	Gas chromatography–mass spectrometry
GFAP	Glial fibrillary acidic protein
GI	Gastrointestinal
GR	Glucocorticoid receptor
HDL	High-density lipoprotein
Hh	Hedgehog
hiPSCs	Human induced pluripotent stem cells
HMG-CoA	Hydroxymethylglutaryl-CoA
IF1	Inhibitory factor 1
iPSCs	Induced pluripotent stem cells
IUGR	Intrauterine growth restriction
LC-MS/MS	Liquid chromatography–tandem mass spectrometry
LOVD	Leiden Open Variation Database
LPS	Lipopolysaccharides
MALDI-MSI	Matrix-assisted laser desorption ionization mass spectrometry imaging
*MAP2*	Microtubule-associated protein 2
MEK	Mitogen-activated protein kinase kinase
MRI	Magnetic resonance imaging
mRPE	Monkey retinal pigment epithelium
mTORC1	Mammalian target of rapamycin complex 1
PT	Pregnanetriol
RPE	Retinal pigment epithelial
RPE-65	Retinal pigment epithelium-specific 65 kDa protein
RTK	Receptor tyrosine kinase
SHH	Sonic Hedgehog
SLOS	Smith–Lemli–Opitz syndrome
SMA	α-smooth muscle actin
Smad2	Mothers against decapentaplegic homolog 2
SREBPs	Sterol regulatory element-binding proteins
SSS	SLOS Severity Score
*SYP*	Synaptophysin
TGF-β	Transforming growth factor beta
THCEO	3β,5α,9α-trihydroxycholest-7-en-6-one
TLR4	Toll-like receptor 4
TOF-SIMS	Time-of-flight secondary ion mass spectrometry
*TUBB3*	Tubulin Beta 3 Class III
TβR-I	TGF-β receptor I
TβR-II	TGF-β receptor II
US	Ultrasound imaging
Wnt	Wingless/integrated
Δ8-isomerase	Steroid 8-isomerase
CVS	Chorionic villus sampling

## References

[1] Prabhu A.V., Luu W., Sharpe L.J., Brown A.J. (2016). Cholesterol-Mediated Degradation of 7-Dehydrocholesterol Reductase Switches the Balance from Cholesterol to Vitamin D Synthesis. J. Biol. Chem..

[2] DHCR7 7-Dehydrocholesterol Reductase—NIH Genetic Testing Registry (GTR)—NCBI. https://www.ncbi.nlm.nih.gov/gtr/genes/1717/.

[3] Lee J.N., Bae S.-H., Paik Y.-K. (2002). Structure and Alternative Splicing of the Rat 7-Dehydrocholesterol Reductase Gene. Biochim. Biophys. Acta.

[4] Witsch-Baumgartner M., Fitzky B.U., Ogorelkova M., Kraft H.G., Moebius F.F., Glossmann H., Seedorf U., Gillessen-Kaesbach G., Hoffmann G.F., Clayton P. (2000). Mutational Spectrum in the Delta7-Sterol Reductase Gene and Genotype-Phenotype Correlation in 84 Patients with Smith-Lemli-Opitz Syndrome. Am. J. Hum. Genet..

[5] Unique Variants in the DHCR7 Gene—Global Variome Shared LOVD. https://databases.lovd.nl/shared/variants/DHCR7/unique#object_id=VariantOnTranscriptUnique%2CVariantOnGenome&id=DHCR7&order=VariantOnGenome%2FClinicalClassifica-tion%2CDESC&search_transcriptid=00000101&search_VariantOnGenome/ClinicalClassification=pathogenic&page_size=100&page=1.

[6] Lazarin G.A., Haque I.S., Evans E.A., Goldberg J.D. (2017). Smith-Lemli-Opitz Syndrome Carrier Frequency and Estimates of in Utero Mortality Rates. Prenat. Diagn..

[7] Park J.E., Lee T., Ha K., Ki C.-S. (2021). Carrier Frequency and Incidence Estimation of Smith-Lemli-Opitz Syndrome in East Asian Populations by Genome Aggregation Database (gnomAD) Based Analysis. Orphanet J. Rare Dis..

[8] Witsch-Baumgartner M., Schwentner I., Gruber M., Benlian P., Bertranpetit J., Bieth E., Chevy F., Clusellas N., Estivill X., Gasparini G. (2008). Age and Origin of Major Smith-Lemli-Opitz Syndrome (SLOS) Mutations in European Populations. J. Med. Genet..

[9] Yılmaz M., Bebek O., Turkyilmaz A. (2024). Smith-Lemli-Opitz Syndrome with Biallelic c.1295A>G (p.Tyr432Cys) Variant in the DHCR7 Gene in a 73-Year-Old Woman: Report of the Oldest Patient. Mol. Syndromol..

[10] Peng Y., Myers R., Zhang W., Alexov E. (2018). Computational Investigation of the Missense Mutations in DHCR7 Gene Associated with Smith-Lemli-Opitz Syndrome. Int. J. Mol. Sci..

[11] LOVD—An Open Source DNA Variation Database System. https://www.lovd.nl/.

[12] (2015). The Deciphering Developmental Disorders Study Large-Scale Discovery of Novel Genetic Causes of Developmental Disorders. Nature.

[13] Pinz H., Pyle L.C., Li D., Izumi K., Skraban C., Tarpinian J., Braddock S.R., Telegrafi A., Monaghan K.G., Zackai E. (2018). De Novo Variants in Myelin Regulatory Factor (MYRF) as Candidates of a New Syndrome of Cardiac and Urogenital Anomalies. Am. J. Med. Genet. A.

[14] Pan X., Tao A.M., Lu S., Ma M., Hannan S.B., Slaugh R., Drewes Williams S., O’Grady L., Kanca O., Person R. (2024). De Novo Variants in FRYL Are Associated with Developmental Delay, Intellectual Disability, and Dysmorphic Features. Am. J. Hum. Genet..

[15] Fitzky B.U., Witsch-Baumgartner M., Erdel M., Lee J.N., Paik Y.-K., Glossmann H., Utermann G., Moebius F.F. (1998). Mutations in the Δ7-Sterol Reductase Gene in Patients with the Smith–Lemli–Opitz Syndrome. Proc. Natl. Acad. Sci. USA.

[16] Karaca E., Harel T., Pehlivan D., Jhangiani S.N., Gambin T., Coban Akdemir Z., Gonzaga-Jauregui C., Erdin S., Bayram Y., Campbell I.M. (2015). Genes That Affect Brain Structure and Function Identified by Rare Variant Analyses of Mendelian Neurologic Disease. Neuron.

[17] Nowaczyk M.J.M., Tan M., Hamid J.S., Allanson J.E. (2012). Smith-Lemli-Opitz Syndrome: Objective Assessment of Facial Phenotype. Am. J. Med. Genet. A.

[18] Cunniff C., Kratz L.E., Moser A., Natowicz M.R., Kelley R.I. (1997). Clinical and Biochemical Spectrum of Patients with RSH/Smith-Lemli-Opitz Syndrome and Abnormal Cholesterol Metabolism. Am. J. Med. Genet..

[19] Ryan A.K., Bartlett K., Clayton P., Eaton S., Mills L., Donnai D., Winter R.M., Burn J. (1998). Smith-Lemli-Opitz Syndrome: A Variable Clinical and Biochemical Phenotype. J. Med. Genet..

[20] Yu H., Lee M.H., Starck L., Elias E.R., Irons M., Salen G., Patel S.B., Tint G.S. (2000). Spectrum of Delta(7)-Dehydrocholesterol Reductase Mutations in Patients with the Smith-Lemli-Opitz (RSH) Syndrome. Hum. Mol. Genet..

[21] Balder J.W., Lansberg P.J., Hof M.H., Wiegman A., Hutten B.A., Kuivenhoven J.A. (2018). Pediatric Lipid Reference Values in the General Population: The Dutch Lifelines Cohort Study. J. Clin. Lipidol..

[22] Chandar V., Gidvani C.H., Gupta A.K., Wilson C.G., Sharma Y.V. (1994). Lipid Profile in Normal Healthy Children. Med. J. Armed Forces India.

[23] Donoghue S.E., Pitt J.J., Boneh A., White S.M. (2018). Smith-Lemli-Opitz Syndrome: Clinical and Biochemical Correlates. J. Pediatr. Endocrinol. Metab. JPEM.

[24] Lee J., Lee H., Oh J., Lim T.H., Kang H., Ko B.S., Cho Y., The Korean Cardiac Arrest Research Consortium KoCARC Investigators (2022). Association between Initial Serum Cholesterol Levels and Outcomes of Patients Hospitalized after Out-of-Hospital Cardiac Arrest: A Retrospective Multicenter Registry Study. J. Pers. Med..

[25] Horwich T.B., Hamilton M.A., Maclellan W.R., Fonarow G.C. (2002). Low Serum Total Cholesterol Is Associated with Marked Increase in Mortality in Advanced Heart Failure. J. Card. Fail..

[26] Selvaraman A., Rahhal S., Bianconi S., Furnary T., Porter F.D. (2025). Assessing Postnatal Mortality in Smith-Lemli-Opitz Syndrome. Am. J. Med. Genet. A.

[27] Jurevics H., Morell P. (1995). Cholesterol for Synthesis of Myelin Is Made Locally, Not Imported into Brain. J. Neurochem..

[28] Goritz C., Mauch D.H., Pfrieger F.W. (2005). Multiple Mechanisms Mediate Cholesterol-Induced Synaptogenesis in a CNS Neuron. Mol. Cell. Neurosci..

[29] Genaro-Mattos T.C., Anderson A., Allen L.B., Korade Z., Mirnics K. (2019). Cholesterol Biosynthesis and Uptake in Developing Neurons. ACS Chem. Neurosci..

[30] Valenza M., Chen J.Y., Di Paolo E., Ruozi B., Belletti D., Ferrari Bardile C., Leoni V., Caccia C., Brilli E., Di Donato S. (2015). Cholesterol-loaded Nanoparticles Ameliorate Synaptic and Cognitive Function in H Untington’s Disease Mice. EMBO Mol. Med..

[31] Lu F., Ferriero D.M., Jiang X. (2022). Cholesterol in Brain Development and Perinatal Brain Injury: More than a Building Block. Curr. Neuropharmacol..

[32] Pang K., Liu C., Tong J., Ouyang W., Hu S., Tang Y. (2022). Higher Total Cholesterol Concentration May Be Associated with Better Cognitive Performance among Elderly Females. Nutrients.

[33] Mistry H., Richardson C.D., Higginbottom A., Ashford B., Ahamed S.U., Moore Z., Matthews F.E., Brayne C., Simpson J.E., Wharton S.B. (2024). Relationships of Brain Cholesterol and Cholesterol Biosynthetic Enzymes to Alzheimer’s Pathology and Dementia in the CFAS Population-Derived Neuropathology Cohort. Neurosci. Res..

[34] Pörn M.I., Tenhunen J., Slotte J.P. (1991). Increased Steroid Hormone Secretion in Mouse Leydig Tumor Cells after Induction of Cholesterol Translocation by Sphingomyelin Degradation. Biochim. Biophys. Acta.

[35] Wang Y., Harding S.V., Thandapilly S.J., Tosh S.M., Jones P.J.H., Ames N.P. (2017). Barley β-Glucan Reduces Blood Cholesterol Levels via Interrupting Bile Acid Metabolism. Br. J. Nutr..

[36] Chatelaine H., Dey P., Mo X., Mah E., Bruno R.S., Kopec R.E. (2021). Vitamin A and D Absorption in Adults with Metabolic Syndrome versus Healthy Controls: A Pilot Study Utilizing Targeted and Untargeted LC-MS Lipidomics. Mol. Nutr. Food Res..

[37] Hintermann B., Holzach P. (1992). Sub-Achilles bursitis--a biomechanical analysis and clinical study. Z. Orthop. Ihre Grenzgeb..

[38] Bitgood M.J., McMahon A.P. (1995). Hedgehog and Bmp Genes Are Coexpressed at Many Diverse Sites of Cell-Cell Interaction in the Mouse Embryo. Dev. Biol..

[39] Xu S., Tang C. (2022). Cholesterol and Hedgehog Signaling: Mutual Regulation and Beyond. Front. Cell Dev. Biol..

[40] Koide T., Hayata T., Cho K.W.Y. (2006). Negative Regulation of Hedgehog Signaling by the Cholesterogenic Enzyme 7-Dehydrocholesterol Reductase. Dev. Camb. Engl..

[41] Honda A., Salen G., Shefer S., Batta A.K., Honda M., Xu G., Tint G.S., Matsuzaki Y., Shoda J., Tanaka N. (1999). Bile Acid Synthesis in the Smith-Lemli-Opitz Syndrome: Effects of Dehydrocholesterols on Cholesterol 7alpha-Hydroxylase and 27-Hydroxylase Activities in Rat Liver. J. Lipid Res..

[42] Bianconi S.E., Conley S.K., Keil M.F., Sinaii N., Rother K.I., Porter F.D., Stratakis C.A. (2011). Adrenal Function in Smith-Lemli-Opitz Syndrome. Am. J. Med. Genet. A.

[43] DeBose-Boyd R.A. (2008). Feedback Regulation of Cholesterol Synthesis: Sterol-Accelerated Ubiquitination and Degradation of HMG CoA Reductase. Cell Res..

[44] Shimano H. (2001). Sterol Regulatory Element-Binding Proteins (SREBPs): Transcriptional Regulators of Lipid Synthetic Genes. Prog. Lipid Res..

[45] Prabhu A.V., Sharpe L.J., Brown A.J. (2014). The Sterol-Based Transcriptional Control of Human 7-Dehydrocholesterol Reductase (DHCR7): Evidence of a Cooperative Regulatory Program in Cholesterol Synthesis. Biochim. Biophys. Acta BBA Mol. Cell Biol. Lipids.

[46] Paik Y.K., Billheimer J.T., Magolda R.L., Gaylor J.L. (1986). Microsomal Enzymes of Cholesterol Biosynthesis from Lanosterol. Solubilization and Purification of Steroid 8-Isomerase. J. Biol. Chem..

[47] Elias E.R., Orth L.E., Li A., Xu L., Jones S.M., Rizzo W.B. (2024). Cholic Acid Increases Plasma Cholesterol in Smith-Lemli-Opitz Syndrome: A Pilot Study. Mol. Genet. Metab. Rep..

[48] Xu L., Korade Z., Rosado D.A., Mirnics K., Porter N.A. (2013). Metabolism of Oxysterols Derived from Nonenzymatic Oxidation of 7-Dehydrocholesterol in Cells. J. Lipid Res..

[49] Xu L., Korade Z., Porter N.A. (2010). Oxysterols from Free Radical Chain Oxidation of 7-Dehydrocholesterol: Product and Mechanistic Studies. J. Am. Chem. Soc..

[50] Xu L., Liu W., Sheflin L.G., Fliesler S.J., Porter N.A. (2011). Novel Oxysterols Observed in Tissues and Fluids of AY9944-Treated Rats: A Model for Smith-Lemli-Opitz Syndrome. J. Lipid Res..

[51] Xu L., Korade Z., Rosado J.D.A., Liu W., Lamberson C.R., Porter N.A. (2011). An Oxysterol Biomarker for 7-Dehydrocholesterol Oxidation in Cell/Mouse Models for Smith-Lemli-Opitz Syndrome. J. Lipid Res..

[52] Xu L., Sheflin L.G., Porter N.A., Fliesler S.J. (2012). 7-Dehydrocholesterol-Derived Oxysterols and Retinal Degeneration in a Rat Model of Smith-Lemli-Opitz Syndrome. Biochim. Biophys. Acta.

[53] Shinkyo R., Xu L., Tallman K.A., Cheng Q., Porter N.A., Guengerich F.P. (2011). Conversion of 7-Dehydrocholesterol to 7-Ketocholesterol Is Catalyzed by Human Cytochrome P450 7A1 and Occurs by Direct Oxidation without an Epoxide Intermediate. J. Biol. Chem..

[54] Huang S.S., Liu I.-H., Chen C.-L., Chang J.-M., Johnson F.E., Huang J.S. (2017). 7-Dehydrocholesterol (7-DHC), But Not Cholesterol, Causes Suppression of Canonical TGF-β Signaling and Is Likely Involved in the Development of Atherosclerotic Cardiovascular Disease (ASCVD). J. Cell. Biochem..

[55] Movassaghi M., Bianconi S., Feinn R., Wassif C.A., Porter F.D. (2017). Vitamin D Levels in Smith-Lemli-Opitz Syndrome. Am. J. Med. Genet. A.

[56] Fitzky B.U., Moebius F.F., Asaoka H., Waage-Baudet H., Xu L., Xu G., Maeda N., Kluckman K., Hiller S., Yu H. (2001). 7-Dehydrocholesterol-Dependent Proteolysis of HMG-CoA Reductase Suppresses Sterol Biosynthesis in a Mouse Model of Smith-Lemli-Opitz/RSH Syndrome. J. Clin. Investig..

[57] Song J., Wang D., Chen H., Huang X., Zhong Y., Jiang N., Chen C., Xia M. (2017). Association of Plasma 7-Ketocholesterol With Cardiovascular Outcomes and Total Mortality in Patients With Coronary Artery Disease. Circ. Res..

[58] Goldenberg A., Wolf C., Chevy F., Benachi A., Dumez Y., Munnich A., Cormier-Daire V. (2004). Antenatal Manifestations of Smith-Lemli-Opitz (RSH) Syndrome: A Retrospective Survey of 30 Cases. Am. J. Med. Genet. A.

[59] Różdżyńska-Świątkowska A., Ciara E., Halat-Wolska P., Krajewska-Walasek M., Jezela-Stanek A. (2021). Anthropometric Characteristics of 65 Polish Smith-Lemli-Opitz Patients. J. Appl. Genet..

[60] Gabor K., Mesev E.V., Madenspacher J., Meacham J., Rai P., Moon S., Wassif C.A., Shaikh S.R., Tucker C.J., Karmaus P. (2024). Sterol Biosynthesis Regulates TLR Signaling and the Innate Immune Response in a Smith-Lemli-Opitz Syndrome Model. J. Clin. Investig..

[61] Schoner K., Witsch-Baumgartner M., Behunova J., Petrovic R., Bald R., Kircher S.G., Ramaswamy A., Kluge B., Meyer-Wittkopf M., Schmitz R. (2020). Smith-Lemli-Opitz Syndrome—Fetal Phenotypes with Special Reference to the Syndrome-specific Internal Malformation Pattern. Birth Defects Res..

[62] Eren E.E., Bilgin N., Urganci N., Kose G. (2021). A Case of Smith-Lemli-Opitz Syndrome Diagnosed with Hypertrophic Pyloric Stenosis. Sisli Etfal Hastan. Tip Bul..

[63] The Lancet Neurology (2022). Rare Diseases: Maintaining Momentum. Lancet Neurol..

[64] Thurm A., Tierney E., Farmer C., Albert P., Joseph L., Swedo S., Bianconi S., Bukelis I., Wheeler C., Sarphare G. (2016). Development, Behavior, and Biomarker Characterization of Smith-Lemli-Opitz Syndrome: An Update. J. Neurodev. Disord..

[65] Sikora D.M., Pettit-Kekel K., Penfield J., Merkens L.S., Steiner R.D. (2006). The near Universal Presence of Autism Spectrum Disorders in Children with Smith-Lemli-Opitz Syndrome. Am. J. Med. Genet. A.

[66] Kaub P.A., Sharp P.C., Ranieri E., Fletcher J.M. (2022). Isolated Autism Is Not an Indication for Smith-Lemli-Opitz Syndrome Biochemical Testing. J. Paediatr. Child Health.

[67] Lalovic A., Merkens L., Russell L., Arsenault-Lapierre G., Nowaczyk M.J.M., Porter F.D., Steiner R.D., Turecki G. (2004). Cholesterol Metabolism and Suicidality in Smith-Lemli-Opitz Syndrome Carriers. Am. J. Psychiatry.

[68] Cenik B., Palka J.M., Thompson B.M., McDonald J.G., Tamminga C.A., Cenik C., Brown E.S. (2022). Desmosterol and 7-Dehydrocholesterol Concentrations in Post Mortem Brains of Depressed People: The Role of Trazodone. Transl. Psychiatry.

[69] Zalewski C.K., Sydlowski S.A., King K.A., Bianconi S., Dang Do A., Porter F.D., Brewer C.C. (2021). Auditory Phenotype of Smith-Lemli-Opitz Syndrome. Am. J. Med. Genet. A.

[70] Miyazaki S., Shimizu N., Miyahara H., Teranishi H., Umeda R., Yano S., Shimada T., Shiraishi H., Komiya K., Katoh A. (2024). DHCR7 Links Cholesterol Synthesis with Neuronal Development and Axonal Integrity. Biochem. Biophys. Res. Commun..

[71] Seger R., Ahn N.G., Posada J., Munar E.S., Jensen A.M., Cooper J.A., Cobb M.H., Krebs E.G. (1992). Purification and Characterization of Mitogen-Activated Protein Kinase Activator(s) from Epidermal Growth Factor-Stimulated A431 Cells. J. Biol. Chem..

[72] Yuan J., Ng W.H., Tian Z., Yap J., Baccarini M., Chen Z., Hu J. (2018). Activating Mutations in MEK1 Enhance Homodimerization and Promote Tumorigenesis. Sci. Signal..

[73] Tomita H., Hines K.M., Herron J.M., Li A., Baggett D.W., Xu L. (2022). 7-Dehydrocholesterol-Derived Oxysterols Cause Neurogenic Defects in Smith-Lemli-Opitz Syndrome. eLife.

[74] Murphy A.R., Haynes J.M., Laslett A.L., Cameron N.R., O’Brien C.M. (2020). Three-Dimensional Differentiation of Human Pluripotent Stem Cell-Derived Neural Precursor Cells Using Tailored Porous Polymer Scaffolds. Acta Biomater..

[75] Dreser N., Madjar K., Holzer A.-K., Kapitza M., Scholz C., Kranaster P., Gutbier S., Klima S., Kolb D., Dietz C. (2020). Development of a Neural Rosette Formation Assay (RoFA) to Identify Neurodevelopmental Toxicants and to Characterize Their Transcriptome Disturbances. Arch. Toxicol..

[76] Song S., Huang H., Guan X., Fiesler V., Bhuiyan M.I.H., Liu R., Jalali S., Hasan M.N., Tai A.K., Chattopadhyay A. (2021). Activation of Endothelial Wnt/β-Catenin Signaling by Protective Astrocytes Repairs BBB Damage in Ischemic Stroke. Prog. Neurobiol..

[77] Choudhary A.K., Jha B. (2008). Imaging Findings of Congenital Glaucoma in Opitz Syndrome. AJNR Am. J. Neuroradiol..

[78] Goodwin H., Brooks B.P., Porter F.D. (2008). Acute Postnatal Cataract Formation in Smith-Lemli-Opitz Syndrome. Am. J. Med. Genet. A.

[79] Rossin E.J., Tsui I., Wong S.C., Hou K.K., Prakhunhungsit S., Blair M.P., Shapiro M.J., Leishman L., Nagiel A., Lifton J.A. (2021). Traumatic Retinal Detachment in Patients with Self-Injurious Behavior: An International Multicenter Study. Ophthalmol. Retina.

[80] López-Cañizares A., Al-Khersan H., Fernandez M.P., Lin B.R., Goduni L., Berrocal A.M. (2023). Smith-Lemli-Optiz Syndrome: Importance of Ophthalmology Referral and Follow-Up. J. Am. Assoc. Pediatr. Ophthalmol. Strabismus.

[81] Pfeffer B.A., Xu L., Porter N.A., Rao S.R., Fliesler S.J. (2016). Differential Cytotoxic Effects of 7-Dehydrocholesterol-Derived Oxysterols on Cultured Retina-Derived Cells: Dependence on Sterol Structure, Cell Type, and Density. Exp. Eye Res..

[82] Pfeffer B.A., Xu L., Fliesler S.J. (2021). Transcriptomic Changes Associated with Loss of Cell Viability Induced by Oxysterol Treatment of a Retinal Photoreceptor-Derived Cell Line: An In Vitro Model of Smith-Lemli-Opitz Syndrome. Int. J. Mol. Sci..

[83] Farkas M.H., Skelton L.A., Ramachandra-Rao S., Au E., Fliesler S.J. (2022). Morphological, Biochemical, and Transcriptomic Characterization of iPSC-Derived Human RPE Cells from Normal and Smith-Lemli-Opitz Syndrome Patients. Mol. Vis..

[84] Sharma A., Kumar G.A., Chattopadhyay A. (2021). Late Endosomal/Lysosomal Accumulation of a Neurotransmitter Receptor in a Cellular Model of Smith-Lemli-Opitz Syndrome. Traffic.

[85] Navyasree K.V., Ramesh S.T., Umasankar P.K. (2023). Cholesterol Regulates Insulin-Induced mTORC1 Signaling. J. Cell Sci..

[86] Genoux A., Pons V., Radojkovic C., Roux-Dalvai F., Combes G., Rolland C., Malet N., Monsarrat B., Lopez F., Ruidavets J.-B. (2011). Mitochondrial Inhibitory Factor 1 (IF1) Is Present in Human Serum and Is Positively Correlated with HDL-Cholesterol. PLoS ONE.

[87] Pires Da Silva J., Wargny M., Raffin J., Croyal M., Duparc T., Combes G., Genoux A., Perret B., Vellas B., Guyonnet S. (2023). Plasma Level of ATPase Inhibitory Factor 1 (IF1) Is Associated with Type 2 Diabetes Risk in Humans: A Prospective Cohort Study. Diabetes Metab..

[88] Delvecchio M., Rapone B., Simonetti S., Fecarotta S., De Carlo G., Favoino E., Loverro M.T., Romano A.M.I., Taurino F., Di Naro E. (2020). Dietary Cholesterol Supplementation and Inhibitory Factor 1 Serum Levels in Two Dizygotic Smith-Lemli-Opitz Syndrome Twins: A Case Report. Ital. J. Pediatr..

[89] Lockhart P.B., Chu V., Zhao J., Gohs F., Thornhill M.H., Pihlstrom B., Mougeot F.B., Rose G.A., Sun Y.-P., Napenas J. (2023). Oral Hygiene and Infective Endocarditis: A Case Control Study. Oral Surg. Oral Med. Oral Pathol. Oral Radiol..

[90] Piekoszewska-Ziętek P., Witt-Porczyk A., Turska-Szybka A., Olczak-Kowalczyk D. (2024). Hygienic Behaviors and Use of Dental Care in Patients with Genetic Syndromes. Sci. Rep..

[91] Kelley R.I. (1995). Diagnosis of Smith-Lemli-Opitz Syndrome by Gas Chromatography/Mass Spectrometry of 7-Dehydrocholesterol in Plasma, Amniotic Fluid and Cultured Skin Fibroblasts. Clin. Chim. Acta Int. J. Clin. Chem..

[92] Honda A., Batta A.K., Salen G., Tint G.S., Chen T.S., Shefer S. (1997). Screening for Abnormal Cholesterol Biosynthesis in the Smith-Lemli-Opitz Syndrome: Rapid Determination of Plasma 7-Dehydrocholesterol by Ultraviolet Spectrometry. Am. J. Med. Genet..

[93] Zimmerman P.A., Hercules D.M., Naylor E.W. (1997). Direct Analysis of Filter Paper Blood Specimens for Identification of Smith-Lemli-Opitz Syndrome Using Time-of-Flight Secondary Ion Mass Spectrometry. Am. J. Med. Genet..

[94] Luo Y., Liu Z., Zeng Y., Zhang Y., Luan Y., Ma L., Chen L., Zou L., Yang J., Huang Z. (2022). A Reliable Tool for Detecting 7-Dehydrocholesterol and Cholesterol in Human Plasma and Its Use in Diagnosis of Smith-Lemli-Opitz Syndrome. J. Sep. Sci..

[95] Westbye A.B., Dizdarevic L.L., Dahl S.R., Asprusten E.A., Bliksrud Y.T., Sandblom A.L., Diczfalusy U., Thorsby P.M., Retterstøl K. (2025). A Sterol Panel for Rare Lipid Disorders: Sitosterolemia, Cerebrotendinous Xanthomatosis and Smith-Lemli-Opitz Syndrome. J. Lipid Res..

[96] Becker S., Röhnike S., Empting S., Haas D., Mohnike K., Beblo S., Mütze U., Husain R.A., Thiery J., Ceglarek U. (2015). LC-MS/MS-Based Quantification of Cholesterol and Related Metabolites in Dried Blood for the Screening of Inborn Errors of Sterol Metabolism. Anal. Bioanal. Chem..

[97] Lin J., Yang X., Wang A., Yang J., Zheng Y., Dong H., Tian Y., Zhang Z., Wang M., Song R. (2024). LC-MS/MS Profiling of Colon Oxysterols and Cholesterol Precursors in Mouse Model of Ulcerative Colitis. J. Chromatogr. A.

[98] Roumain M., Muccioli G.G. (2025). Development and Application of an LC-MS/MS Method for the Combined Quantification of Oxysterols and Bile Acids. J. Lipid Res..

[99] Yasuda Y., Tateishi N., Shimoda T., Satoh S., Ogitani E., Fujita S. (2004). Relationship between S100beta and GFAP Expression in Astrocytes during Infarction and Glial Scar Formation after Mild Transient Ischemia. Brain Res..

[100] Esgul N., Orhan Varoglu A., Baysal B. (2025). Association of Gray and White Matter Volumes, Clinical Features, Neurofilament Light Chain, and Glial Fibrillary Acidic Protein in Relapsing-Remitting Multiple Sclerosis. Acta Radiol..

[101] Abdelhak A., Foschi M., Abu-Rumeileh S., Yue J.K., D’Anna L., Huss A., Oeckl P., Ludolph A.C., Kuhle J., Petzold A. (2022). Blood GFAP as an Emerging Biomarker in Brain and Spinal Cord Disorders. Nat. Rev. Neurol..

[102] Biberthaler P., Musaelyan K., Krieg S., Meyer B., Stimmer H., Zapf J., Von Matthey F., Chandran R., Marino J.A., Beligere G. (2021). Evaluation of Acute Glial Fibrillary Acidic Protein and Ubiquitin C-Terminal Hydrolase-L1 Plasma Levels in Traumatic Brain Injury Patients with and without Intracranial Lesions. Neurotrauma Rep..

[103] Fukuyama R., Izumoto T., Fushiki S. (2001). The Cerebrospinal Fluid Level of Glial Fibrillary Acidic Protein Is Increased in Cerebrospinal Fluid from Alzheimer’s Disease Patients and Correlates with Severity of Dementia. Eur. Neurol..

[104] Zhu N., Santos-Santos M., Illán-Gala I., Montal V., Estellés T., Barroeta I., Altuna M., Arranz J., Muñoz L., Belbin O. (2021). Plasma Glial Fibrillary Acidic Protein and Neurofilament Light Chain for the Diagnostic and Prognostic Evaluation of Frontotemporal Dementia. Transl. Neurodegener..

[105] Norgren N., Sundström P., Svenningsson A., Rosengren L., Stigbrand T., Gunnarsson M. (2004). Neurofilament and Glial Fibrillary Acidic Protein in Multiple Sclerosis. Neurology.

[106] Freel B.A., Kelvington B.A., Sengupta S., Mukherjee M., Francis K.R. (2022). Sterol Dysregulation in Smith-Lemli-Opitz Syndrome Causes Astrocyte Immune Reactivity through Microglia Crosstalk. Dis. Model. Mech..

[107] Tcw J., Qian L., Pipalia N.H., Chao M.J., Liang S.A., Shi Y., Jain B.R., Bertelsen S.E., Kapoor M., Marcora E. (2022). Cholesterol and Matrisome Pathways Dysregulated in Astrocytes and Microglia. Cell.

[108] Luke R.A., Cawley N.X., Rahhal S., Selvaraman A., Thurm A., Wassif C.A., Porter F.D. (2024). Elevated Cerebrospinal Fluid Glial Fibrillary Acidic Protein Levels in Smith-Lemli-Opitz Syndrome. Mol. Genet. Metab..

[109] Saraste M., Bezukladova S., Matilainen M., Sucksdorff M., Kuhle J., Leppert D., Airas L. (2021). Increased Serum Glial Fibrillary Acidic Protein Associates with Microstructural White Matter Damage in Multiple Sclerosis: GFAP and DTI. Mult. Scler. Relat. Disord..

[110] Aladia A.H., Hamdan S., Alkheder A. (2024). First Documented Case of Smith-Lemli-Opitz Syndrome in Syria: Clinical Presentation, Diagnosis, and Experimental Management with Simvastatin. Oxf. Med. Case Rep..

[111] Lee R.W.Y., Conley S.K., Gropman A., Porter F.D., Baker E.H. (2013). Brain Magnetic Resonance Imaging Findings in Smith-Lemli-Opitz Syndrome. Am. J. Med. Genet. A.

[112] Li A., Xu L. (2023). MALDI-IM-MS Imaging of Brain Sterols and Lipids in a Mouse Model of Smith-Lemli-Opitz Syndrome. BioRxiv Prepr. Serv. Biol..

[113] Haas D., Haege G., Hoffmann G.F., Burgard P. (2013). Prenatal Presentation and Diagnostic Evaluation of Suspected Smith-Lemli-Opitz (RSH) Syndrome. Am. J. Med. Genet. A.

[114] Waye J.S., Eng B., Nowaczyk M.J.M. (2007). Prenatal Diagnosis of Smith-Lemli-Opitz Syndrome (SLOS) by DHCR7 Mutation Analysis. Prenat. Diagn..

[115] Lopez J., Mikaelian I., Gonzalo P. (2013). Amniotic Fluid Glial Fibrillary Acidic Protein (AF-GFAP), a Biomarker of Open Neural Tube Defects. Prenat. Diagn..

[116] Shackleton C.H.L., Marcos J., Palomaki G.E., Craig W.Y., Kelley R.I., Kratz L.E., Haddow J.E. (2007). Dehydrosteroid Measurements in Maternal Urine or Serum for the Prenatal Diagnosis of Smith-Lemli-Opitz Syndrome (SLOS). Am. J. Med. Genet. A.

[117] Rohanizadegan M., Sacharow S. (2018). Desmosterolosis Presenting with Multiple Congenital Anomalies. Eur. J. Med. Genet..

[118] Kroes I., Janssens S., Defoort P. (2014). Ultrasound Features in Trisomy 13 (Patau Syndrome) and Trisomy 18 (Edwards Syndrome) in a Consecutive Series of 47 Cases. Facts Views Vis. ObGyn.

[119] Luo Y., Zhang C., Ma L., Zhang Y., Liu Z., Chen L., Wang R., Luan Y., Rao Y. (2022). Measurement of 7-Dehydrocholesterol and Cholesterol in Hair Can Be Used in the Diagnosis of Smith-Lemli-Opitz Syndrome. J. Lipid Res..

[120] Pappu A.S., Steiner R.D., Connor S.L., Flavell D.P., Lin D.S., Hatcher L., Illingworth D.R., Connor W.E. (2002). Feedback Inhibition of the Cholesterol Biosynthetic Pathway in Patients with Smith-Lemli-Opitz Syndrome as Demonstrated by Urinary Mevalonate Excretion. J. Lipid Res..

[121] Wassif C.A., Kratz L., Sparks S.E., Wheeler C., Bianconi S., Gropman A., Calis K.A., Kelley R.I., Tierney E., Porter F.D. (2017). A Placebo-Controlled Trial of Simvastatin Therapy in Smith-Lemli-Opitz Syndrome. Genet. Med..

[122] Björkhem I. (2006). Crossing the Barrier: Oxysterols as Cholesterol Transporters and Metabolic Modulators in the Brain. J. Intern. Med..

[123] Gosselet F., Saint-Pol J., Fenart L. (2014). Effects of Oxysterols on the Blood-Brain Barrier: Implications for Alzheimer’s Disease. Biochem. Biophys. Res. Commun..

[124] Gao Y., Ye S., Tang Y., Tong W., Sun S. (2023). Brain Cholesterol Homeostasis and Its Association with Neurodegenerative Diseases. Neurochem. Int..

[125] Sikora D.M., Ruggiero M., Petit-Kekel K., Merkens L.S., Connor W.E., Steiner R.D. (2004). Cholesterol Supplementation Does Not Improve Developmental Progress in Smith-Lemli-Opitz Syndrome. J. Pediatr..

[126] Maciejak A., Leszczynska A., Warchol I., Gora M., Kaminska J., Plochocka D., Wysocka-Kapcinska M., Tulacz D., Siedlecka J., Swiezewska E. (2013). The Effects of Statins on the Mevalonic Acid Pathway in Recombinant Yeast Strains Expressing Human HMG-CoA Reductase. BMC Biotechnol..

[127] Ballout R.A., Livinski A., Fu Y.-P., Steiner R.D., Remaley A.T. (2022). Statins for Smith-Lemli-Opitz Syndrome. Cochrane Database Syst. Rev..

[128] Gonzales E., Matarazzo L., Franchi-Abella S., Dabadie A., Cohen J., Habes D., Hillaire S., Guettier C., Taburet A.-M., Myara A. (2018). Cholic Acid for Primary Bile Acid Synthesis Defects: A Life-Saving Therapy Allowing a Favorable Outcome in Adulthood. Orphanet J. Rare Dis..

[129] Korade Z., Xu L., Harrison F.E., Ahsen R., Hart S.E., Folkes O.M., Mirnics K., Porter N.A. (2014). Antioxidant Supplementation Ameliorates Molecular Deficits in Smith-Lemli-Opitz Syndrome. Biol. Psychiatry.

[130] Koczok K., Horváth L., Korade Z., Mezei Z.A., Szabó G.P., Porter N.A., Kovács E., Mirnics K., Balogh I. (2021). Biochemical and Clinical Effects of Vitamin E Supplementation in Hungarian Smith-Lemli-Opitz Syndrome Patients. Biomolecules.

[131] Ertugrul G., Yankol Y., Mecit N., Kirimlioglu H., Kanmaz T., Acarli K., Kalayoglu M. (2022). Liver Transplant and Improvements in Cholesterol Biosynthesis Defects: A Case Report of Smith-Lemli-Opitz Syndrome. Exp. Clin. Transplant. Off. J. Middle East Soc. Organ Transplant..

[132] Pasta S., Akhile O., Tabron D., Ting F., Shackleton C., Watson G. (2015). Delivery of the 7-Dehydrocholesterol Reductase Gene to the Central Nervous System Using Adeno-Associated Virus Vector in a Mouse Model of Smith-Lemli-Opitz Syndrome. Mol. Genet. Metab. Rep..

[133] Phillips C., Parkinson A., Namsrai T., Chalmers A., Dews C., Gregory D., Kelly E., Lowe C., Desborough J. (2024). Time to Diagnosis for a Rare Disease: Managing Medical Uncertainty. A Qualitative Study. Orphanet J. Rare Dis..

[134] Fisch G.S. (2017). Whither the Genotype-phenotype Relationship? An Historical and Methodological Appraisal. Am. J. Med. Genet. C Semin. Med. Genet..

[135] Ciara E., Nowaczyk M., Witsch-Baumgartner M., Malunowicz E., Popowska E., Jezela-Stanek A., Piotrowicz M., Waye J., Utermann G., Krajewska-Walasek M. (2004). *DHCR7* Mutations and Genotype–Phenotype Correlation in 37 Polish Patients with Smith–Lemli–Opitz Syndrome. Clin. Genet..

[136] Díaz-Santiago E., Jabato F.M., Rojano E., Seoane P., Pazos F., Perkins J.R., Ranea J.A.G. (2020). Phenotype-Genotype Comorbidity Analysis of Patients with Rare Disorders Provides Insight into Their Pathological and Molecular Bases. PLoS Genet..

[137] Trujillano D., Oprea G., Schmitz Y., Bertoli-Avella A.M., Abou Jamra R., Rolfs A. (2017). A Comprehensive Global Genotype–Phenotype Database for Rare Diseases. Mol. Genet. Genom. Med..

[138] Schmidt A., Danyel M., Grundmann K., Brunet T., Klinkhammer H., Hsieh T.-C., Engels H., Peters S., Knaus A., Moosa S. (2024). Next-Generation Phenotyping Integrated in a National Framework for Patients with Ultrarare Disorders Improves Genetic Diagnostics and Yields New Molecular Findings. Nat. Genet..

[139] Rillig F., Grüters A., Schramm C., Krude H. (2022). The Interdisciplinary Diagnosis of Rare Diseases—Results of the Translate-NAMSE Project. Dtsch. Ärztebl. Int..

[140] Lin J., De Rezende V.L., De Aguiar Da Costa M., De Oliveira J., Gonçalves C.L. (2023). Cholesterol Metabolism Pathway in Autism Spectrum Disorder: From Animal Models to Clinical Observations. Pharmacol. Biochem. Behav..

[141] Abedalthagafi M., Bawazeer S., Fawaz R.I., Heritage A.M., Alajaji N.M., Faqeih E. (2023). Non-Invasive Prenatal Testing: A Revolutionary Journey in Prenatal Testing. Front. Med..

[142] Fernandez N., Escobar R., Zarante I. (2016). Craniofacial Anomalies Associated with Hypospadias. Description of a Hospital Based Population in South America. Int. Braz J Urol Off. J. Braz. Soc. Urol..

[143] Joss S.K., Paterson W., Donaldson M.D.C., Tolmie J.L. (2002). Cleft Palate, Hypotelorism, and Hypospadias: Schilbach-Rott Syndrome. Am. J. Med. Genet..

[144] Wadman E., Fernandes E., Muss C., Powell-Hamilton N., Wojcik M.H., Madden J.A., Carreon C.K., Clark R.D., Stenftenagel A., Chikalard K. (2023). A Novel Syndrome Associated with Prenatal Fentanyl Exposure. Genet. Med. Open.

[145] Elias E.R., Irons M.B., Hurley A.D., Tint G.S., Salen G. (1997). Clinical Effects of Cholesterol Supplementation in Six Patients with the Smith-Lemli-Opitz Syndrome (SLOS). Am. J. Med. Genet..

[146] Starck L., Lövgren-Sandblom A., Björkhem I. (2002). Simvastatin Treatment in the SLO Syndrome: A Safe Approach?. Am. J. Med. Genet..

[147] Calvo P.L., Brunati A., Spada M., Romagnoli R., Corso G., Parenti G., Rossi M., Baldi M., Carbonaro G., David E. (2014). Liver Transplantation in Defects of Cholesterol Biosynthesis: The Case of Lathosterolosis. Am. J. Transplant..

[148] Fehr A., Prütz F. (2023). Rare Diseases: A Challenge for Medicine and Public Health. J. Health Monit..

[149] Depping M.K., Uhlenbusch N., von Kodolitsch Y., Klose H.F.E., Mautner V.-F., Löwe B. (2021). Supportive Care Needs of Patients with Rare Chronic Diseases: Multi-Method, Cross-Sectional Study. Orphanet J. Rare Dis..

[150] Gutenbrunner C., Schiller J., Goedecke V., Lemhoefer C., Boekel A. (2022). Screening of Patient Impairments in an Outpatient Clinic for Suspected Rare Diseases-A Cross-Sectional Study. Int. J. Environ. Res. Public Health.

[151] Begic N., Begic Z., Begic E. (2021). Smith-Lemli-Opitz Syndrome: Bosnian and Herzegovinian Experience. Balk. J. Med. Genet. BJMG.

[152] Tang S., Yuan K., Chen L. (2022). Molecular Biomarkers, Network Biomarkers, and Dynamic Network Biomarkers for Diagnosis and Prediction of Rare Diseases. Fundam. Res..

[153] Bai J.P.F., Barrett J.S., Burckart G.J., Meibohm B., Sachs H.C., Yao L. (2013). Strategic Biomarkers for Drug Development in Treating Rare Diseases and Diseases in Neonates and Infants. AAPS J..

[154] Ayoglu B., Chaouch A., Lochmüller H., Politano L., Bertini E., Spitali P., Hiller M., Niks E.H., Gualandi F., Pontén F. (2014). Affinity Proteomics within Rare Diseases: A BIO-NMD Study for Blood Biomarkers of Muscular Dystrophies. EMBO Mol. Med..

[155] Samuel J.P., Wootton S.H., Tyson J.E. (2023). N-of-1 Trials: The Epitome of Personalized Medicine?. J. Clin. Transl. Sci..

[156] Ying L., Matabosch X., Serra M., Watson B., Shackleton C., Watson G. (2014). Biochemical and Physiological Improvement in a Mouse Model of Smith–Lemli–Opitz Syndrome (SLOS) Following Gene Transfer with AAV Vectors. Mol. Genet. Metab. Rep..

[157] Horgan C., Watts K., Ram D., Rust S., Hutton R., Jones S., Wynn R. (2023). A Retrospective Cohort Study of Libmeldy (Atidarsagene Autotemcel) for MLD: What We Have Accomplished and What Opportunities Lie Ahead. JIMD Rep..

